# Wine Spoilage Control: Impact of Saccharomycin on *Brettanomyces bruxellensis* and Its Conjugated Effect with Sulfur Dioxide

**DOI:** 10.3390/microorganisms9122528

**Published:** 2021-12-07

**Authors:** Patrícia Branco, Rute Coutinho, Manuel Malfeito-Ferreira, Catarina Prista, Helena Albergaria

**Affiliations:** 1 Unit of Bioenergy and Biorefinery, LNEG, Estrada do Paço do Lumiar, 22, 1649-038 Lisboa, Portugal; 2 Linking Landscape, Environment, Agriculture and Food (LEAF), Associated Laboratory TERRA, Instituto Superior de Agronomia, University of Lisbon, Tapada da Ajuda, 1349-017 Lisboa, Portugal; anarute.coutinho@gmail.com (R.C.); mmalfeito@isa.ulisboa.pt (M.M.-F.)

**Keywords:** biocontrol, wine-spoilage, biocides, preservatives, wine fermentation

## Abstract

The yeast *Brettanomyces bruxellensis* is one of the most dangerous wine contaminants due to the production of phenolic off-flavors such as 4-ethylphenol. This microbial hazard is regularly tackled by addition of sulfur dioxide (SO_2_). Nevertheless, *B. bruxellensis* is frequently found at low levels (ca 10^3^ cells/mL) in finished wines. Besides, consumers health concerns regarding the use of sulfur dioxide encouraged the search for alternative biocontrol measures. Recently, we found that *Saccharomyces cerevisiae* secretes a natural biocide (saccharomycin) that inhibits the growth of different *B. bruxellensis* strains during alcoholic fermentation. Here we investigated the ability of *S. cerevisiae* CCMI 885 to prevent *B. bruxellensis* ISA 2211 growth and 4-ethylphenol production in synthetic and true grape must fermentations. Results showed that *B. bruxellensis* growth and 4-ethylphenol production was significantly inhibited in both media, although the effect was more pronounced in synthetic grape must. The natural biocide was added to a simulated wine inoculated with 5 × 10^2^ cells/mL of *B. bruxellensis*, which led to loss of culturability and viability (100% dead cells at day-12). The conjugated effect of saccharomycin with SO_2_ was evaluated in simulated wines at 10, 12, 13 and 14% (*v*/*v*) ethanol. Results showed that *B. bruxellensis* proliferation in wines at 13 and 14% (*v*/*v*) ethanol was completely prevented by addition of 1.0 mg/mL of saccharomycin with 25 mg/L of SO_2_, thus allowing to significantly reduce the SO_2_ levels commonly used in wines (150–200 mg/L).

## 1. Introduction

The indigenous microbiota of grape musts includes an immense variety of microorganisms that can grow and ferment sugars [1,2]. Nowadays, most wine fermentations are conducted by selected yeast starters, which are mainly composed of *Saccharomyces cerevisiae* strains, due to their fast fermentation rates and ability to survive in the harsh environmental conditions of wine [3,4,5]. Although *S. cerevisiae* strains usually dominate alcoholic fermentations, some microorganisms such as lactic and acetic acid bacteria and yeasts like *Dekkera*/*Brettanomyces bruxellensis* may remain in finished wines and proliferate under certain conditions (e.g., oxygen and/or sugars availability), spoiling the wine [6,7].

Amongst wine contaminants, *B. bruxellensis* (anamorphic form) and its ascospore-forming type *D. bruxellensis* (teleomorphic form) is considered the most dangerous spoilage microorganism and has been isolated from almost every wine-producing area of the world [8,9,10]. In red wines, but also in some white wines, *B. bruxellensis* produces volatile phenols such as 4-ethylphenol and 4-ethylguaiacol, which have characteristic aromas described as ‘‘barnyard-like’’ or ‘‘horsey-like” [8,11]. Due to the wine spoilage potential of *B. bruxellensis*, control measures are required to prevent its growth and metabolic activity.

Addition of sulfur dioxide (SO_2_) is the most common preservative practice in winemaking [12] since this chemical is highly toxic to most wine microbial contaminants [1,13] Besides, SO_2_ has several other properties, acting in wines as an antioxidant [14] antioxidasic [12] and antimicrobial agent [15,16]. SO_2_ can be added at different stages of the winemaking process: directly to musts, after alcoholic and malolactic fermentation, during wine aging, and at bottling. However, the use of SO_2_ can be harmful to humans since exposure to sulfites can cause a range of adverse reactions such as dermatitis, urticaria, hypotension, abdominal pain, and diarrhea [17,18]. Besides, several studies [19,20] reported the existence of *B. bruxellensis* strains resistant to SO_2_ at the levels legally allowed in finished wines (150–200 mg/L) [21]. The increasing concerns regarding the excessive use of chemical additives in foodstuffs have raised interest on finding alternative bioprotection methods [22,23,24].

In recent years, several killer toxins secreted by different yeasts (e.g., *Saccharomyces eubayanus* and *Candida pyralidae*) have been suggested as biocontrol agents of *B. bruxellensis* under winemaking conditions [25,26]. In previous work [27,28,29,30], we found that several *S. cerevisiae* strains secrete a natural biocide, which we named saccharomycin, that is active against wine-related non-*Saccharomyces* yeasts, including *B. bruxellensis*, as well as lactic acid bacteria. Saccharomycin was found to be composed of antimicrobial peptides (AMPs) derived from the glycolytic enzyme glyceraldehyde-3-phosphate dehydrogenase, and its minimal inhibitory concentration against six *B. bruxellensis* strains (i.e., ISA 1649, ISA 1700, ISA 1791, ISA 2104, ISA 2116, and ISA 2211) was found to vary between 1–2 mg/mL [28]. Besides, *S. cerevisiae* CCMI 885 exerted an antagonistic effect against all the above-mentioned *B.* *bruxellensis* strains during synthetic grape juice mixed-culture fermentations [29]. In this context, saccharomycin presents itself as a promising biopreservative that might be used in alternative and/or in conjugation with SO_2_.

The aim of the present work was to evaluate the effectiveness of saccharomycin to prevent *B. bruxellensis* growth and 4-ethylphenol production in wine fermentations, as well as to assess its conjugated effect with SO_2_ against *B. bruxellensis* proliferation in finished wines, thus allowing to reduce the chemical levels presently used in winemaking.

## 2. Materials and Methods

### 2.1. Strains and Inoculums

In this work we used the *Saccharomyces cerevisiae* strain CCMI 885 (Culture Collection of Industrial Microorganisms, LNEG, Lisbon, Portugal) and *Brettanomyces bruxellensis* strain ISA 2211, from Instituto Superior de Agronomia (ISA, University of Lisbon, Portugal). Inoculums of yeast strains were prepared by transferring one YEPD-agar slant into 50 mL of YEPD medium (10 g/L yeast extract, 20 g/L peptone and 20 g/L glucose) and incubating the cultures at 30 °C, under agitation (150 rpm) during 16 h for *S. cerevisiae* and 72 h for *B. bruxellensis*.

### 2.2. Growth Media

Alcoholic fermentations were performed with *B. bruxellensis* in single-culture and in mixed-culture with *S. cerevisiae* using a Synthetic-Grape Must (SGM) and a True-Grape Must (TGM). The SGM, contained 110 g/L of D-glucose (Merck, Darmstadt, Germany), 110 g/L of D-fructose (Merck, Darmstadt, Germany), 6.0 g/L of tartaric acid (Sigma-Aldrich, St. Louis, MO, USA), 3.0 g/L malic acid (Sigma-Aldrich, St. Louis, MO, USA), 0.5 g/L of citric acid (Sigma-Aldrich, St. Louis, MO, USA), 1.7 g/L of yeast nitrogen base w/out amino acids (Difco Laboratories, Franklin Lakes, NJ, USA), 2.0 g/L of casamino acids (Merck, Darmstadt, Germany), 0.2 g/L CaCl_2_ (Merck, Darmstadt, Germany), 0.8 g/L of arginine-HCl (Sigma-Aldrich, St. Louis, MO, USA), 1.0 g/L of proline (Sigma-Aldrich, St. Louis, MO, USA), and 0.1 g/L of tryptophan (Sigma-Aldrich, St. Louis, MO, USA), pH 3.5. The SGM was prepared as described in [31]. The TGM was prepared using 2012 vintage white grapes (*Vitis vinifera* L. cv. Alvarinho, Viosinho and Encruzado) collected from an experimental vineyard of Instituto Superior de Agronomia (Lisbon, Portugal). The grapes were frozen at −70 °C and stored until the beginning of the assay (approximately 6 months). Grapes were manually crushed, and the obtained grape juice was centrifuged at 10,000× *g* for 15 min (twice) and filtered sequentially through the following pore-size membrane filters (Millipore): 8.0 µm; 1.2 µm; 0.45 µm (twice). Finally, the cleared juice was filter-sterilized twice again using 0.22 µm membrane and the pH adjusted to 3.5 with ortho-phosphoric acid.

### 2.3. Synthetic-Grape Must (SGM) and True-Grape Must (TGM) Fermentations Performed with B. bruxellensis in Single- and in Mixed-Culture with S. cerevisiae

SGM and TGM fermentations were performed in 500 mL flasks containing 300 mL of each medium (supplemented with 10 mg/L of p-coumaric acid) that were inoculated with 5 × 10^4^ cells/mL of *B. bruxellensis* (strain ISA 2211) in single-culture fermentations and with 5 × 10^4^ cells/mL of each yeast species (i.e., of *S. cerevisiae* and *B. bruxellensis*) in the mixed-culture fermentations. All fermentations (i.e., single- and mixed-culture SGM and TGM fermentations) were carried-out in duplicates and incubated at 25 °C, under slow agitation (80 rpm). Daily samples were taken from each flask to determine yeasts culturability, *B. bruxellensis* viability, as well as sugars consumption and ethanol and 4-ethylphenol production.

### 2.4. Analysis of Growth

#### 2.4.1. Culturability

Culturability of *S. cerevisiae* and *B. bruxellensis* was determined by the classical plating method. Briefly, 100 µL of samples were plated onto YEPD-agar plates, after appropriate dilution (decimal serial dilution method). Plates were incubated at 30 °C (Vertical Incubator, Infors HT, Anjou, QC, Canada) and the number of Colonies Forming Units (CFU) enumerated after 2–6 days. In the mixed-culture fermentations, CFU counts of *B. bruxellensis* were obtained on 0.01% (*w*/*v*) cycloheximide YEPD-agar plates and the CFU counts of *S. cerevisiae* as the difference between total CFU counts (corresponding to *S. cerevisiae* plus *B. bruxellensis*) on YEPD-agar plates and the CFU counts of *B. bruxellensis*. The detection limit of the CFU method was 1 CFU/mL for results given in Section 3.1 and Section 3.2.1, since 1000 µL were directly inoculated onto YEPD-agar plates for samples where no colonies were detected in 100 µL.

#### 2.4.2. Viability

Viability (live/dead) of *B. bruxellensis* cells in single-culture fermentations was determined by directly applying the Live/Dead staining (LDS) procedure, as described below. For mixed-culture samples, PI-stained cells were then subjected to the Fluorescence In Situ Hybridization (FISH) method, the so-called LDS-FISH method, using the protocol described in [32]. The species-specific FISH-probe used to hybridize *B. bruxellensis* cells (26S-D.brux. 5.1) was designed by [33] and comprises the following oligonucleotide sequence: 5′-CTTACTCAAATCCCTCCGGT-3′. This FISH-probe was synthesized and labelled with the fluorochrome Fluorescein Isothiocyanate (FITC) at the 5′-end by demand of external services (STAB VIDA, Lisbon, Portugal).

LDS procedure: Briefly, 1 mL of culture medium was collected daily from single- and mixed-culture fermentations and cells were concentrated by centrifugation at 10,000× *g* for 10 min. The pellet was then washed with Bovine Serum Albumin (BSA)-saline solution (0.25% BSA *w*/*v*, 0.1% NaCl *w*/*v*) by gently pipetting up and down several times. Afterwards, the cell suspension was centrifuged again at 10,000× *g* for 10 min and resuspended in 100–1000 μL of BSA-saline solution, depending on the cellular density. Then, 10 μL of Propidium Iodide (PI, supplied by Life Technologies, Carlsbad, CA, USA) working solution (5 μg/mL) was mixed with 100 μL of cellular suspension (ca 10^6^ cells/mL) and incubated for 10–20 min at room temperature without light.

LDS-FISH method: After applying the LDS procedure above-described, the PI-stained cellular suspension was centrifuged for 5 min at 5000× *g*, the pellet was washed once with 1× phosphate-buffered saline solution (PBS) and then incubated with 4% (*v*/*v*) of paraformaldehyde for 4 h at 4 °C under agitation. Afterwards, fixed cells (approx. 10^6^ cells) were centrifuged for 2 min at 10,000× *g* and hybridized in 45 μL of hybridization buffer (0.9 M sodium chloride, 0.01 % *w*/*v* sodium dodecyl sulfate, 20 mM Tris-HCl and 5 % *v*/*v* formamide) together with 5 μL of FITC labelled probe (50 ng/μL). Incubation was performed at 46 °C for 3 h. Subsequently, the cell suspension was centrifuged again (5 min at 10,000× *g*) and cells resuspended in 100 μL of washing solution (25 mM Tris/HCl and 0.5 M NaCl). This mixture was incubated for 30 min at 48 °C. Before enumeration, the previous suspension was again centrifuged, and cells resuspended in 100 μL of 1× PBS.

**Figure 1:**
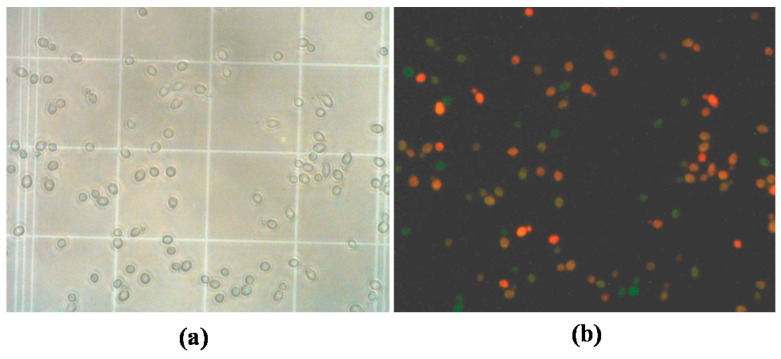
Microscopic visualization (objective amplification, 40×) of cells in a mixed-culture sample after applying of the LDS-FISH procedure. (**a**) Cells observed in the bright-field; (**b**) cells observed in the U-MWB filter.

Quantification of live/dead cells: after applying the LDS or the LDS-FISH treatment, approximately 5 μL of each cell suspension was mixed with 5 μL of Vecta Shield (Vector Laboratories, Burlingame, CA, USA), spotted onto a Neubauer chamber and cells enumerated using an epifluorescence microscope (Olympus BX-60, Tokyo, Japan). Total cells were visualized in the bright field of the microscope and fluorescent cells in the U-MWB filter. Figure 1 shows LDS-FISH treated cells from a mixed-culture sample, visualized in the bright field (Figure 1a) and in the U-MWB filter (Figure 1b), where green cells correspond to live *B. bruxellensis* cells (FISH-hybridized cells/non-PI-stained), orange/yellow cells correspond to dead *B. bruxellensis* cells (FISH-hybridized/PI-stained) and red cells correspond to dead *S. cerevisiae* cells (not FISH-hybridized/PI-stained).

### 2.5. Quantification of Sugars and Ethanol by High Performance Liquid Chromatography

Sugars (glucose and fructose) and ethanol were quantified by High Performance Liquid Chromatography (HPLC), using an HPLC system (Merck Hitachi, Darmstadt, Germany) equipped with a refractive index detector (L-7490, Merck Hitachi, Darmstadt, Germany). Fermentation samples were filtered through 0.22 µm Millipore filters (Merck Millipore, Algés, Portugal) and then injected (20 µL) in a Sugar-Pack column (Waters Hitachi, Milford, CT, USA). Samples were eluted using as mobile phase CaEDTA (50 mg/L) at 90 °C, with a flow-rate of 0.5 mL/min. All samples were analysed in duplicate. Glucose, fructose, and ethanol standards at concentrations of 15, 7.5 and 3.75 g/L were used to construct calibration curves.

### 2.6. Quantification of 4-Ethylphenol by Gas-Chromatography

The concentration of 4-ethylphenol (4-EP) produced by *B. bruxellensis* during single- and mixed-culture SGM and TGM fermentations was quantified by gas-chromatography (GC) using filtered (0.22 µm Millipore filters) samples that were first frozen at −18 °C in 15 mL Falcon tubes (Orange Scientific, Braine-L’Alleud, Belgium) and kept frozen until use. 4-EP was quantified using the protocol described in [9,34]. The volatile phenol (4-EP) was extracted using ether-hexan from a 5 mL sample with pH adjusted to 8.0 with NaOH. The volatile 4-EP was separated by collecting the organic phase of the mixture. The quantification was achieved by gas chromatography using 3,4-dimethylphenol as internal standard. A GC-FID (Varian CP-3800 series, Walnut Creek, CA, USA) with a capillary column Factor-Four (internal diameter 0.25 mm, length 15 m, film thickness 0.25µm) was used. The injector was run in split less mode, at 230 °C and the volume of injection was 2 µL. The detector temperature was set to 250 °C. Hydrogen was used as gas carrier at a flow rate of 0.1 mL/min. The oven was initially set at 50 °C, then the temperature was raised to 215 °C at a 10 °C/min rate and finally increased up to 250 °C at a rate of 20 °C/min. Calibration curves were constructed using 4-EP standards with concentration values ranging from 0–10 mg/L.

### 2.7. Production and Purification of Saccharomycin

The natural biocide (saccharomycin) secreted by *S. cerevisiae* (strain CCMI 885) was obtained from a SGM-fermentation performed at 25 °C without agitation for 7 days. The 7 day-old fermented broth was filtered through 0.22 μm Millipore membranes (Merck Millipore, Algés, Portugal) and the supernatant was first ultrafiltrated using 10 kDa centrifugal units (Vivaspin 15R, Sartorius, Germany) and then the permeate (<10 kDa) was concentrated (40-fold) in similar centrifugal units equipped with 2 kDa membranes. Finally, 100 μL of this concentrated peptidic fraction (2–10 kDa) was fractionated by size-exclusion chromatography using a Superdex-Peptide column (10/300 GL, GE Healthcare, Buckinghamshire, UK). The HPLC system was equipped with an UV-detector (Merck Hitachi, Darmstadt, Germany) and samples were eluted with ammonium acetate 0.1 M at a flow rate of 0.7 mL/min. The chromatographic pick with retention-time 26–27 min, previously found to contain saccharomycin [28,30] was collected, lyophilized, and stored frozen at −20 °C until required.

### 2.8. Effectiveness of the Natural Biocide to Prevent B. bruxellensis Growth in Wine

300 mL of TGM were fermented by *S. cerevisiae* at 25 °C without agitation for 20 days. Then, the fermented broth was filtered through 0.22 µm Millipore filters (Merck Millipore, Algés, Portugal) and the 2–10 kDa peptidic fraction of this cell-free supernatant was ultrafiltrated and concentrated (40-fold) as described in the previous sub-section. The 20-day-old fermented supernatant (pH 3.5), containing 118 g/L ethanol and no sugars, was supplemented with 8 g/L of fructose to simulate a wine with residual sugars that allow microbial growth, i.e., the “simulated wine”. 2 mL of the above-mentioned peptidic fraction was added to this “simulated wine” that was then inoculated with 5 × 10^2^ CFU/mL of *B. bruxellensis* (strain ISA 2211). A control-assay was performed in the same “simulated wine” but without addition of the 2–10 kDa peptidic fraction, which was used as Control. Culture-assays were incubated at 25 °C without agitation. Culturability of *B. bruxellensis* was followed by plate counts (CFU/mL), as described in Section 2.4.1, and viability by the LDS procedure described in Section 2.4.2.

### 2.9. Conjugated Effect of Saccharomycin with Sulfur Dioxide (SO_2_) on B. bruxellensis Culturability

Simulated wines were prepared using the SGM medium (pH 3.5) mentioned in Section 2.2, modified in its sugars solution to contain just 4.5 g/L of fructose. Ethanol was added to this modified-SGM to obtain simulated wines with 10%, 12%, 13% and 14% (*v*/*v*), respectively, with final pH values of 3.5. Each simulated wine was artificially contaminated with 5 × 10^3^ cells/mL of *B. bruxellensis* in a final volume of 300 µL. First, the inhibitory effects of ethanol and SO_2_ were analyzed in separate, i.e., simulated wines without SO_2_ but with 10%, 12%, 13% and 14% (*v*/*v*) ethanol, respectively, were used to evaluate the ethanol effect on *B. bruxellensis* growth; simulated wines without ethanol but with 25, 50, 100 and 150 mg/L of potassium metabisulfite (PMB) (Sigma-Aldrich, Missouri, EUA) (concentrations equivalent to 0.16, 0.33, 0.66 and 1 mg/L of molecular SO_2_, at pH 3.5) were used to assess the SO_2_ effect on *B. bruxellensis* growth. Then, the synergistic effect of SO_2_ with ethanol was tested using simulated wines at all ethanol levels (i.e., at 10%, 12%, 13% and 14% (*v*/*v*) ethanol), each of them supplemented with 25, 50, 100 and 150 mg/L of PMB (Sigma-Aldrich, Missouri, EUA). Finally, the conjugated effect of saccharomycin (obtained as described in Section 2.7) with SO_2_ was evaluated on *B. bruxellensis* growth using the simulated wines at all ethanol levels (i.e., at 10%, 12%, 13% and 14% (*v*/*v*) ethanol), each of them supplemented with 0.25, 0.5 and 1 mg/mL of saccharomycin together with PMB at 25 and 50 mg/L, respectively. All growth-assays were performed in triplicates in 96 well-microplates and incubated in a Multiskan-GO spectrophotometer (Thermo-Fisher Scientific Inc., Waltham, MA, USA) at 30 °C, under strong agitation. Cell growth was followed by optical density measurements (at 590 nm) in a Microplate Reader (Dinex Technologies Inc., Chantilly, VA, USA) and by CFU counts. For CFU counts, 10 µL of samples were taken and after appropriate dilution (decimal serial dilution method) 100 µL were plated onto YEPD-agar plates, as described in the Section 2.4.1. Whenever colonies could not be detected in agar-plates inoculated with diluted samples, 100 µL of sample were directly plated onto YEPD-agar plates. Thus, the detection limit of the CFU method for results presented in Section 3.2.2 was 10 CFU/mL.

### 2.10. Statistical Analyses

The minimum significant difference between results presented in Table 1 and in figures was calculated to allow comparison of mean values, as described by Fry et al. [35]. To check the assumption of equal variances the Levene’s test was used and then, one way ANOVA (if the variances were equal) or Welch tests (if the variances were unequal) were applied to determine the significance of the difference between means. The statistical analysis was performed in Microsoft Excel.

## 3. Results

### 3.1. Synthetic-Grape Must (SGM) and True-Grape Must (TGM) Fermentations Performed with B. bruxellensis in Single- and in Mixed-Cultures with S. cerevisiae

**Figure 2:**
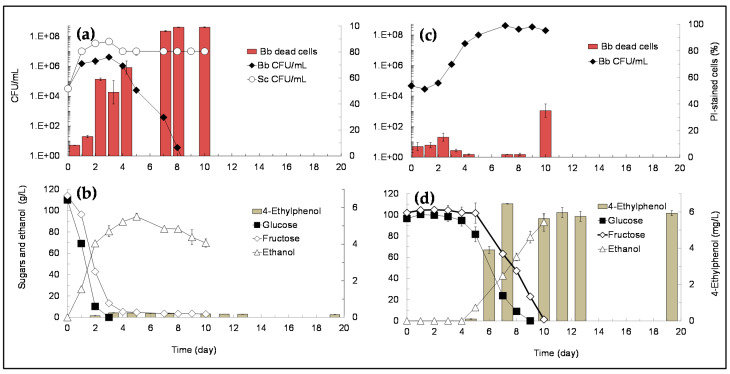
Yeast growth and metabolic profiles during SGM fermentations. (**a**) Culturability (CFU/mL) of *S. cerevisiae* (Sc) and *B. bruxellensis* (Bb), and viability of Bb (% dead cells) during mixed-culture fermentation; (**b**) sugars consumption, and ethanol and 4-ethylphenol production during mixed-culture fermentation; (**c**) Culturability and viability of Bb during single-culture fermentation; (**d**) sugars consumption, and ethanol and 4-ethylphenol production during Bb single-culture fermentation. Values presented correspond to means (± SD) of duplicate measurements of two independent biological experiments.

Metabolic and yeasts growth profiles during SGM fermentations performed with *B. bruxellensis* in single-culture and in mixed-culture with *S. cerevisiae* are represented in Figure 2. During mixed-culture fermentations (Figure 2a,b) *S. cerevisiae* increased its cell density from an initial cell density of 5 × 10^4^ CFU/mL up to 4 × 10^7^ CFU/mL after 3 days, remaining at about 10^7^ CFU/mL until the end of fermentation (day-10), while *B. bruxellensis* grew during the first 3 days (from 5 × 10^4^ CFU/mL up to 4 × 10^6^ CFU/mL) but then began to die-off, decreasing its culturability in the next 5 days (to 4 CFU/mL at day-8) (Figure 2a). The loss of culturability of *B. bruxellensis* during the mixed-culture fermentation was accompanied by an increase of the number of dead cells (PI-stained cells) (Figure 2a) that represented 99% of the population at day-8. Since the number of culturable cells is extremely low at day-8 (4 CFU/mL) and 99% of the total cell population was dead, the percentage of viable but non-culturable (VBNC) cells should be less than 1%. Conversely, during the single-culture fermentation (Figure 2c,d) *B. bruxellensis* culturability increased from 5 × 10^4^ CFU/mL at day-0 up to 4 × 10^8^ CFU/mL at day-7, remaining at about 10^8^ CFU/mL until the end of fermentation (10 days) (Figure 2c). During the single-culture fermentation (Figure 2c,d) *B. bruxellensis* cell viability (live/dead cells) correlated with its culturability, since the number of viable cells (non-PI-stained cells) remained high throughout fermentation (ranging from 92–98% during the first 8 days) and decreased to only 65% at the end of fermentation (day-10) (Figure 2c), when sugars were already completely consumed (Figure 2d). Metabolic profiles (i.e., sugars consumption, and ethanol and 4-ethylphenol production) during the mixed-culture fermentation (Figure 2b) show that sugars (glucose and fructose) were almost completely consumed within the first 5 days (4.7 g/L of residual fructose), when ethanol attained its highest level (92 g/L), and 4-ethylphenol was produced in very low amounts, attaining a maximal concentration of 0.25 mg/L at day-3. The negligible levels of 4-ethylphenol produced during the mixed-culture fermentation (Figure 2b) correlate with the loss of *B. bruxellensis* viability (Figure 2a). On the contrary, during *B. bruxellensis* single-culture fermentation (Figure 2c,d) sugars were consumed at a much slower rate (the same amount of sugars was consumed only after 10 days) and ethanol attained its highest concentration (93 g/L) after 10 days (Figure 2d), showing that *B. bruxellensis* metabolism is much slower than that of *S. cerevisiae*. Regarding 4-ethylphenol, results show that this phenolic compound was produced at significantly higher levels in the single-culture fermentation (Figure 2d) attaining 6.44 mg/L at day-7, what can be explained by the high culturability of *B. bruxellensis* during this fermentation (Figure 2c). Comparing the culturability/viability profiles of *B. bruxellensis* in single-culture fermentation (Figure 2c) with that in mixed-culture fermentation (Figure 2a), it becomes clear that *S. cerevisiae* exerted a strong antagonistic effect against *B. bruxellensis* growth and 4-ethylphenol production.

**Figure 3:**
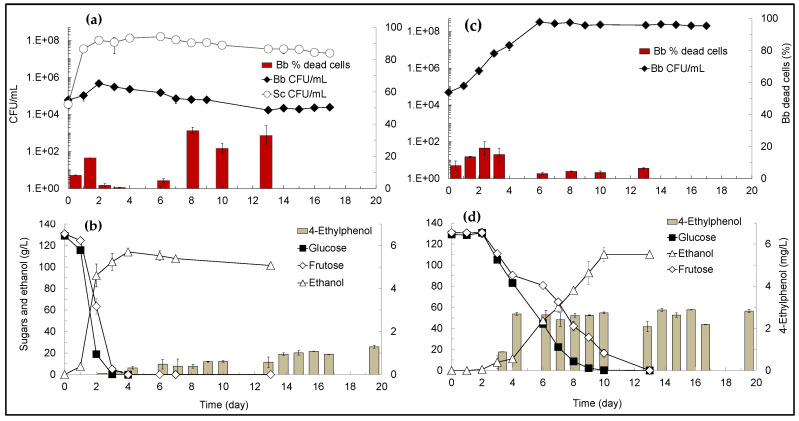
Yeast growth and metabolic profiles during TGM fermentations. (**a**) Culturability (CFU/mL) of *S. cerevisiae* (Sc) and *B. bruxellensis* (Bb), and viability of Bb (% dead cells) during mixed-culture fermentation; (**b**) sugars consumption, and ethanol and 4-ethylphenol production during mixed-culture fermentation; (**c**) Culturability and viability of Bb during single-culture fermentation; (**d**) sugars consumption, and ethanol and 4-ethylphenol production during Bb single-culture fermentation. Values presented correspond to means (± SD) of duplicate measurements of two independent biological experiments.

To check if the antagonistic effect exerted by *S. cerevisiae* against *B. bruxellensis* would also be effective in TGM, mixed- and single-culture fermentations were performed at the same growth conditions. Yeasts growth and metabolic profiles during the mixed- and single-culture TGM-fermentations are shown in Figure 3. Results show that *S. cerevisiae* exerted an antagonistic effect against *B. bruxellensis* also in the TGM-fermentation (Figure 3a,b), although the effect was less pronounced than in the SGM-fermentation. In fact, while *B. bruxellensis* completely lost its culturability and viability within 8 days (<10 CFU/mL and >99% dead-cells) in the mixed-culture SGM-fermentation (Figure 2a), in the TGM-fermentation *B. bruxellensis* was able to grow in the first 2 days (up to 4.7 × 10^5^ CFU/mL) but then its culturability decreased to 1.7 × 10^4^ CFU/mL at day-13, as well as its viability (from 92% at day-0 to 77% at day-13) (Figure 3a). In the single-culture TGM-fermentation (Figure 3c,d), *B. bruxellensis* was able to grow in the first 6 days, increasing its cell density from 5 × 10^4^ CFU/mL at day-0 to 3 × 10^8^ CFU/mL at day-6 and keeping this value (10^8^ CFU/mL) for 17 days, while dead cells remained at low numbers (ranging 6–15% of PI-stained cells) (Figure 3c). Once again, one can conclude that *S. cerevisiae* inhibited *B. bruxellensis* metabolism since a much lower level of 4-ethylphenol (1.3 mg/L) was produced during the mixed-culture TGM-fermentation (Figure 3b) by comparison with 2.82 mg/L of 4-ethylphenol produced during the single-culture fermentation (Figure 3d).

### 3.2. Biopreservative Potential of Saccharomycin in Wine

#### 3.2.1. Effect of Saccharomycin on *B. bruxellensis* Culturability and Viability

**Figure 4:**
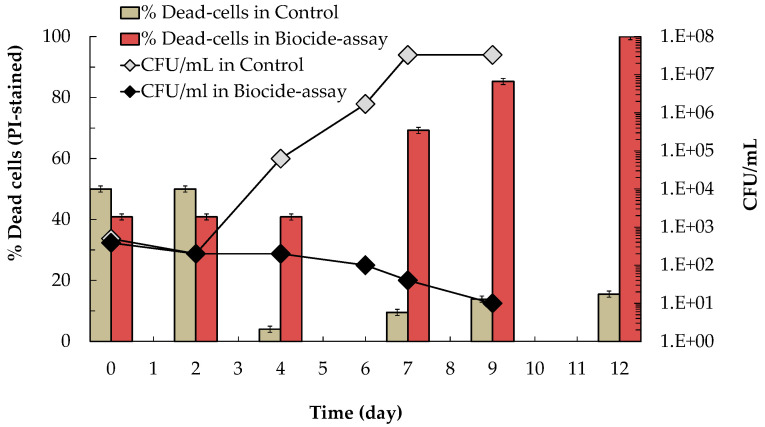
Effect of saccharomycin on the culturability (CFU/mL) and viability (PI-stained cells) of *B. bruxellensis* during the biocide assay (simulated wine with 1 mg/mL of saccharomycin), and in the respective control-assay (simulated wine without saccharomycin). The detection limit of the CFU method was 1 CFU/mL. Values presented correspond to means (± SD) of duplicate measurements of two independent biological experiments.

To evaluate the effectiveness of the natural biocide (saccharomycin) to prevent *B. bruxellensis* proliferation in wine, a simulated wine (118 g/L of ethanol and 8 g/L of residual fructose, pH 3.5) supplemented with 1 mg/mL of the peptidic fraction containing the natural biocide was artificially contaminated with 5 × 10^2^ cells/mL of *B. bruxellensis*. Culturability (CFU/mL) and viability (PI-staining) profiles of *B. bruxellensis* in the biocide-assay and in the control-assay (without biocide) are shown in Figure 4. Results show that while in the control-assay *B. bruxellensis* was able to grow after the second day of inoculation, attaining 3.3 × 10^7^ CFU/mL at day-7, in the biocide-assay *B. bruxellensis* culturability continuously decreased upon inoculation attaining a cell density of 10 CFU/mL at day-9. The loss of *B. bruxellensis* culturability in the biocide-assay was accompanied by an increase of the percentage of dead cells that reached 85% at day-9 and 100% at day-12, while in the control-assay, viability of *B. bruxellensis* remained high even after 12 days (15% of cells dead) (Figure 4).

#### 3.2.2. Conjugated Effect of Saccharomycin with Sulfur Dioxide (SO_2_)

**Figure 5:**
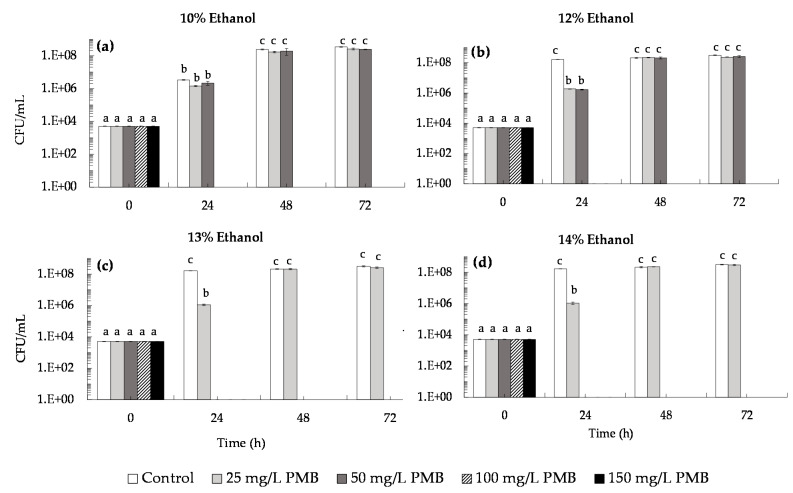
Inhibitory effect of potassium metabisulfite (PMB) at 0 (control), 25, 50, 100 and 150 mg/L PMB on *B. bruxellensis* culturability (CFU/mL) in simulated wine inoculated with 5 × 10^3^ CFU/mL of *B. bruxellensis*. (**a**) Simulated wine with 10% (*v*/*v*) ethanol; (**b**) simulated wine with 12% (*v*/*v*) ethanol; (**c**) simulated wine with 13% (*v*/*v*) ethanol; (**d**) simulated wine with 14% (*v*/*v*) ethanol). Values presented correspond to means (± SD) of duplicate measurements of three independent biological experiments. Different letters located over the error bars indicate significantly different values (*p* < 0.05).

The single effect of ethanol and potassium metabisulfite (PMB) on *B. bruxellensis* growth was evaluated in simulated wines (pH 3.5), artificially contaminated with 5 × 10^3^ cells/mL of *B. bruxellensis*. Results (Table 1) showed that *B. bruxellensis* was able to grow in the presence of 10%, 12%, 13% and 14% (*v*/*v*) of ethanol, reaching 3 × 10^8^ CFU/mL after 72 h. Likewise, SO_2_ at 25, 50, 100 and 150 mg/L of PMB was not able to inhibit growth of *B. bruxellensis* in simulated wines without ethanol, with cultures reaching similar cell density levels (i.e., ca 10^8^ CFU/mL) after 72 h (Table 1). The combined effect of ethanol (10%, 12%, 13% and 14% (*v*/*v*)) with PMB (25, 50, 100 and 150 mg/L of PMB) was also assessed. Results (Figure 5) revealed that in simulated wines at 10% and 12% (*v*/*v*) ethanol, *B. bruxellensis* growth was completely inhibited by 100 and 150 mg/L of PMB (i.e., 0.66 and 1.0 mg/mL of molecular SO_2_), respectively (Figure 5a,b), whereas in simulated wines at 13% and 14% (*v*/*v*) ethanol, *B. bruxellensis* was only able to proliferate in the presence of 25 mg/L of PMB (0.16 mg/mL of molecular SO_2_) (Figure 5c,d). Our results are in accordance with the probabilistic model developed by Sturm et al. [36] for *B. bruxellensis* growth as a function of pH, ethanol and free SO_2_, which predicts that *B. bruxellensis* is not able to grow in a simulated wine with 50 mg/L of free SO_2_ (ca 150 mg/mL of PMB) when conjugated with ethanol levels between 10% and 15% (*v*/*v*) and pH values between 3.3 to 4.1.

**Figure 6:**
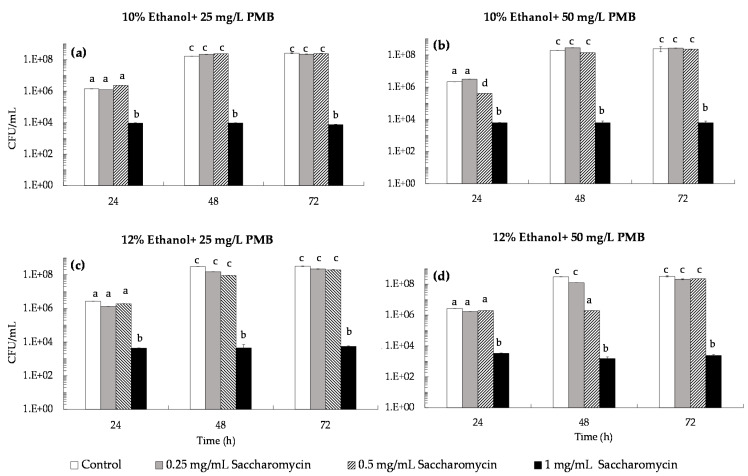
Conjugated effect of saccharomycin (0.25, 0.5 and 1.0 mg/mL) with potassium metabisulfite (PMB) on *B. bruxellensis* culturability (CFU/mL) in simulated wine inoculated with 5 × 10^3^ CFU/mL of *B. bruxellensis*. (**a**) Simulated wine at 10% ethanol and 25 mg/L PMB; (**b**) simulated wine at 10% ethanol with 50 mg/L PMB; (**c**) simulated wine at 12% ethanol with 25 mg/L PMB; (**d**) simulated wine at 12% ethanol with 50 mg/L PMB. Values presented correspond to means (± SD) of duplicate measurements of three independent biological experiments. Different letters located over the error bars indicate significantly different values (*p* < 0.05).

**Figure 7:**
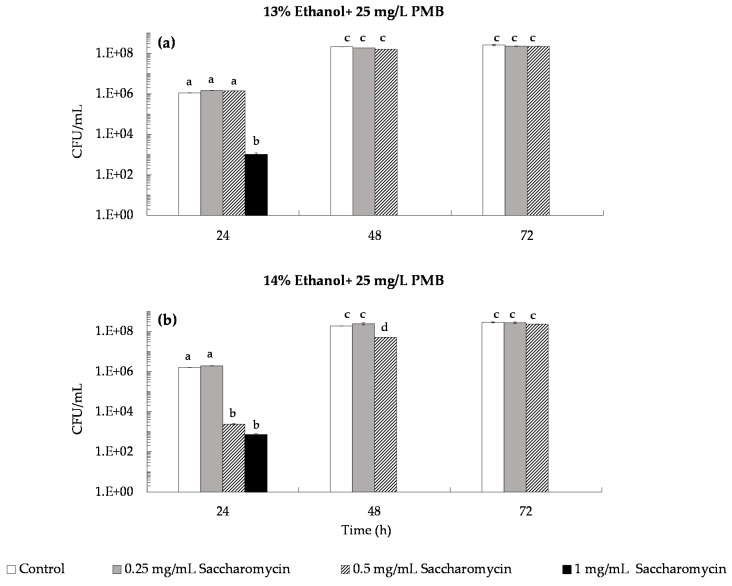
Conjugated effect of saccharomycin (0.25, 0.5 and 1.0 mg/mL) with 25 mg/L of potassium metabisulfite (PMB) on *B. bruxellensis* culturability (CFU/mL) in simulated wines inoculated with 5 × 10^3^ CFU/mL of *B. bruxellensis*. (**a**) Simulated wine at 13% ethanol; (**b**) simulated wine at 14% ethanol. Values presented correspond to means (± SD) of duplicate measurements of three independent biological experiments. Different letters located over the error bars indicate significantly different values (*p* < 0.05).

The inhibitory effect of saccharomycin was tested at concentrations of 0.25, 0.5 and 1.0 mg/mL in simulated wines at 10% and 12% (*v*/*v*) of ethanol together with 25 mg/L of PMB (Figure 6a,c) and 50 mg/L of PMB (Figure 6b,d). Results showed that in both wines inhibition of *B. bruxellensis* growth was only achieved with addition of 1.0 mg/mL of saccharomycin together with SO_2_ at both 25 and 50 mg/mL PMB (Figure 6a–c). However, even addition of 1.0 mg/mL saccharomycin was not sufficient to induce total loss of *B. bruxellensis* culturability with cultures remaining at ca 10^3^–10^4^ CFU/mL after 72 h (Figure 6a–c).In simulated wines at 13% and 14% (*v*/*v*) ethanol, the inhibitory effect of saccharomycin (at 0.25, 0.5 and 1.0 mg/mL) together with 25 mg/mL of PMB (Figure 7a,b) revealed that 0.5 mg/mL of saccharomycin prevented *B. bruxellensis* growth above 5 × 10^3^ CFU/mL in the first 24 h in the simulated wine at 14% (*v*/*v*) ethanol, while addition of 1.0 mg/mL saccharomycin induced loss of *B. bruxellensis* culturability (to less than 10 CFU/mL) in both simulated wines (i.e., wines at 13% and 14% (*v*/*v*) ethanol) (Figure 7a,b). This demonstrate that 1.0 mg/mL of saccharomycin together with 25 mg/L of PMB (0.16 mg/mL of molecular SO_2_) is sufficient to reduce *B. bruxellensis* culturability below 10 CFU/mL within 48 h in wines at 13% and 14% (*v*/*v*) ethanol (Figure 7).

## 4. Discussion

In previous work we found that *S. cerevisiae* secretes a natural biocide (saccharomycin) during alcoholic fermentation that mediates the early death of *Hanseniaspora guilliermondii* in mixed-culture alcoholic fermentations [28] and inhibits the growth of wine-related non-*Saccharomyces* yeasts, including *B. bruxellensis* [28,30]. The effect of saccharomycin was evaluated against the growth of six *B. bruxellensis* strains (i.e., ISA 1649, ISA 1700, ISA 1791, ISA 2104, ISA 2116 and ISA 2211) in YEPD medium (at pH 3.5) demonstrating to it inhibits all those strains, although the minimal inhibitory concentration varied amongst strains, from 1–2 mg/mL [28]. Besides, *S. cerevisiae* CCMI 885 also demonstrated to exert an antagonistic effect against all the six *B. bruxellensis* strains during synthetic grape must (SGM) mixed-culture fermentations [29]. Those results [28,29,30,37] strongly suggested that saccharomycin is, at least in part, responsible for the antagonism exerted by *S. cerevisiae* against *B. bruxellensis* during mixed-culture alcoholic fermentations. In fact, results obtained in the present work (Figure 2 and Figure 3) support that assumption, since *B. bruxellensis* rapidly lost its culturability (i.e., from 4.1 × 10^6^ CFU/mL at day-3 to 4 CFU/mL at day-8 in SGM and from 4.7 × 10^5^ CFU/mL at day-3 to 1.7 × 10^4^ CFU/mL at day-13 in TGM) during the mixed-culture fermentations (Figure 2a and Figure 3a) but kept its culturability at high levels (ca 10^8^ CFU/mL) during the single-culture fermentations (Figure 2c and Figure 3c), namely after total sugars exhaustion (Figure 3c). Thus, neither nutrients depletion nor oxygen requirements can explain the early death of *B. bruxellensis* during mixed-culture fermentations.

In the previous studies [28,29,30] alcoholic fermentations were performed in SGM, and not in TGM, and we did not investigate the impact of the antagonistic effect of *S. cerevisiae* on the metabolism of *B. bruxellensis*, namely on the production of 4-ethylphenol. Thus, in the present work we evaluated and compared the inhibitory effect of *S. cerevisiae* against *B. bruxellensis* growth and 4-ethylphenol production during synthetic- and true-grape must fermentations. Results showed that the antagonistic effect exerted by *S. cerevisiae* on *B. bruxellensis* growth and 4-ethylphenol production was higher in SGM (Figure 2) than in TGM fermentations (Figure 3). The difference observed might be due to partial inactivation of saccharomycin by its adsorption by proteins in suspension present in true-grape musts [12], amongst other factors such as micronutrients in TGM that may favor *B. bruxellensis* growth. Even though, in TGM-fermentations, the presence of *S. cerevisiae* cells at high cell density (i.e., above 10^7^ cells/mL) significantly prevented *B. bruxellensis* growth and reduced the levels of 4-ethyphenol produced (Figure 3a,b), compared with profiles exhibited by *B. bruxellensis* in single-culture fermentation (Figure 3c,d). However, during mixed-culture TGM fermentations, *B. bruxellensis* was able to produce 1.3 mg/L of 4-ethylphenol, which is a concentration higher than the level perceived as negative in red wines, i.e., 0.62 mg/L [12]. This led us to conclude that to fully prevent spoilage of wine by *B. bruxellensis* strains, it would be necessary to add this natural biopreservative (saccharomycin) to wines. Indeed, our results (Figure 4) showed that addition of 1 mg/mL of saccharomycin to a simulated wine (at 15% (*v*/*v*) of ethanol), artificially contaminated with 5 × 10^2^ CFU/mL of *B. bruxellensis*, was sufficient to induce total death of *B. bruxellensis* in 12 days. These results agree well with our previous findings, which showed that in YEPD medium with 30 g/L ethanol (at pH 3.5) the minimum inhibitory concentrations (MIC) of saccharomycin against several *B. bruxellensis* strains ranged 1–2 mg/mL [28].

According to the European regulation (EC) n° 606/2009, the maximum concentration of sulfur dioxide that can be added to red and white wines (with residual sugars lower than 5 g/L and 10–14% (*v*/*v*) ethanol) is 150 mg/L and 200 mg/L, respectively. However, nowadays, reduction of chemical additives in food manufacturing processes is a societal demand, mainly due to health concerns. In addition, global warming has led to production of wines with increased pH values, which reduces SO_2_ antimicrobial efficiency [21]. These concerns are pressing winemakers to search for new preservation practices that can substitute or complement the antimicrobial effect of SO_2_, allowing them to reduce the levels of SO_2_ added to wine [22,23,24]. In the present work, we evaluated the preservation effect of the natural biocide, saccharomycin, against *B. bruxellensis*, added to simulated wines alone (Figure 4) and in conjugation with SO_2_ (Figure 6 and Figure 7). First, we evaluated the sensitivity of *B. bruxellensis* ISA 2211 towards SO_2_ alone in simulated wines at 10, 12, 13 and 14% ethanol. Results (Figure 5) showed that in simulated wines at 10% and 12% (*v*/*v*) ethanol, *B. bruxellensis* was only able to grow for SO_2_ levels lower than 100 mg/L PMB (0.66 mg/L of molecular SO_2_) and in wines with 13 and 14% (*v*/*v*) ethanol for SO_2_ levels lower than 50 mg/L PMB (0.33 mg/L of molecular SO_2_). Our results agree with those of Barata et al. [10] who tested the effect of PMB against several strains of *B. bruxellensis* in red wines, showing that most of the *B. bruxellensis* strains evaluated, including the strain tested in the present study (i.e., strain ISA 2211), were not able to grow with 100–150 mg/L of PMB. They are also in agreement with results reported by Avramova et al. [38] that classified *B. bruxellensis* ISA 2211 as sensitive towards SO_2_ and belonging to the CBS 2499-like group, where most strains are not able to grow with 0.6 mg/L of molecular SO_2,_ i.e., with ca 100 mg/L of PMB. Finally, the conjugated effect of saccharomycin (0.25, 0.5 and 1.0 mg/mL) with SO_2_ was evaluated in simulated wines with PMB concentrations that allowed growth in each of the simulated wine, i.e., 25 and 50 mg/L PMB for wines at 10 and 12% (*v*/*v*) ethanol and 25 mg/L for wines at 13 and 14% (*v*/*v*) ethanol. Results showed that addition of 1 mg/mL of saccharomycin to wines at 10% and 12% (*v*/*v*) ethanol, prevented *B. bruxellensis* growth above the inoculation level (i.e., 5 × 10^3^ CFU/mL) both for wines with 25 and 50 mg/L PMB (Figure 6). In simulated wines at 13% and 14% (*v*/*v*) ethanol, addition of 1 mg/mL of saccharomycin allowed to reduce the SO_2_ levels to 25 mg/L PMB (i.e., ca 0.16 mg/L molecular SO_2_), induing the loss of *B. bruxellensis* culturability to less than 10 CFU/mL (Figure 7).

Thus, our work shows that saccharomycin is a promising wine biopreservative that allows reducing the levels of SO_2_ usually used in winemaking. However, the present results should be considered as preliminary results since they were obtained at micro-scale growth conditions and not under true wine production conditions. Besides, the impact of other parameters, such as the initial level of *B bruxellensis* contamination, wine pH and cells adaptation to ethanol, on the inhibitory efficiency of saccharomycin should also be further assessed.

## Figures and Tables

**Table 1:** Independent effect of ethanol and sulfur dioxide on the culturability (CFU/mL) of *B. bruxellensis* (strain ISA 2211) inoculated in simulated wines (modified-SGM) with 10, 12, 13 and 14% (*v*/*v*) of ethanol, pH 3.5, and in the same modified-SGM without ethanol but with 25, 50, 100 and 150 mg/L of potassium metabisulfite (PMB) that correspond to concentrations of molecular SO_2_ of 0.16, 0.33, 0.66 and 1 mg/L, respectively. Values presented correspond to means ( ± SD) of duplicate measurements of three independent biological experiments. Different letters located before the CFU/mL indicate significantly different values (*p* < 0.05).

*B. bruxellensis* Culturability (CFU/mL)									
Time (h)	Control	Ethanol (% *v*/*v*)				PMB (mg/L)			
	-	10	12	13	14	25	50	100	150
0	^a^ (5.0 ± 0.2) × 10^3^	^a^ (5.0 ± 0.2) × 10^3^	^a^ (5.0 ± 0.2) × 10^3^	^a^ (5.0 ± 0.2) × 10^3^	^a^ (5.0 ± 0.2) × 10^3^	^a^ (5.0 ± 0.2) × 10^3^	^a^ (5.0 ± 0.2) × 10^3^	^a^ (5.0 ± 0.2) × 10^3^	^a^ (5.0 ± 0.2) × 10^3^
24	^b^ (1.8 ± 0.2) × 10^8^	^c^ (3.3 ± 0.1) × 10^6^	^c^ (2.7 ± 0.5) × 10^6^	^d^ (2.4 ± 0.1) × 10^6^	^e^ (1.6 ± 0.1) × 10^6^	^f^ (4.0 ± 0.1) × 10^5^	^f^ (3.7 ± 0.1) × 10^5^	^g^ (3.0 ± 0.1) × 10^4^	^h^ (7.0 ± 1.2) × 10^3^
48	^b^ (1.9 ± 0.2) × 10^8^	^i^ (3.4 ± 0.2) × 10^8^	^i^ (3.0 ± 0.1) × 10^8^	^i^ (2.6 ± 0.1) × 10^8^	^b^ (1.9 ± 0.2) × 10^8^	^j^ (3.5 ± 0.1) × 10^7^	^j^ (3.9 ± 0.1) × 10^7^	^k^ (2.9 ± 0.5) × 10^7^	^l^ (1.6 ± 0.6) × 10^7^
72	^i^ (3.1 ± 0.1) × 10^8^	^i^ (3.2 ± 0.2) × 10^8^	^i^ (3.3 ± 0.1) × 10^8^	^i^ (3.3 ± 0.3) × 10^8^	^i^ (2.8 ± 0.2) × 10^8^	^i^ (3.5 ± 0.3) × 10^8^	^i^ (3.2 ± 0.1) × 10^8^	^i^ (2.8 ± 0.5) × 10^8^	^i^ (2.8 ± 0.1) × 10^8^

## Data Availability

All data reported in this study is included in the manuscript.
